# Construction of a nomogram for preoperative prediction of the risk of lymph node metastasis in early gastric cancer

**DOI:** 10.3389/fsurg.2022.986806

**Published:** 2023-01-06

**Authors:** Zitao Liu, Huakai Tian, Yongshan Huang, Yu Liu, Feilong Zou, Chao Huang

**Affiliations:** ^1^ Department of Gastrointestinal Surgery, The Second Affiliated Hospital of Nanchang University, Nanchang, China; ^2^ Department of Gastrointestinal Surgery, The First Affiliated Hospital of Nanchang University, Nanchang, China

**Keywords:** early gastric cancer, lymph node metastasis, systemic inflammation response index, nomogram, risk

## Abstract

**Background:**

The status of lymph node metastasis (LNM) in patients with early gastric cancer (EGC) is particularly important for the formulation of clinical treatment. The purpose of this study was to construct a nomogram to predict the risk of LNM in EGC before operation.

**Methods:**

Univariate analysis and logistic regression analysis were used to determine the independent risk factors for LNM. The independent risk factors were included in the nomogram, and the prediction accuracy, discriminant ability and clinical practicability of the nomogram were evaluated by the receiver operating characteristic curve (ROC), calibration curve and clinical decision curve (DCA), and 100 times ten-fold cross-validation was used for internal validation.

**Results:**

33 (11.3%) cases of AGC were pathologically confirmed as LNM. In multivariate analysis, T stage, presence of enlarged lymph nodes on CT examination, carbohydrate antigen 199 (CA199), undifferentiated histological type and systemic inflammatory response index (SIRI) were risk factors for LNM. The area under the ROC curve of the nomogram was 0.86, the average area under the ROC curve of the 100-fold ten-fold cross-validation was 0.85, and the *P* value of the Hosmer-Lemeshow test was 0.60. In addition, the clinical decision curve, net reclassification index (NRI) and Integrated Discriminant Improvement Index (IDI) showed that the nomogram had good clinical utility.

**Conclusions:**

We found that SIRI is a novel biomarker for preoperative prediction of LNM in EGC, and constructed a nomogram for preoperative prediction of the risk of LNM in EGC, which is helpful for the formulation of the clinical treatment strategies.

## Introduction

Gastric cancer ranks fifth in incidence among malignant tumors, which is one of the leading causes of cancer-related deaths (1). With the popularity of gastric cancer screening programs, the promotion of health awareness and the improvement of endoscopic equipment, more and more early gastric cancers have been diagnosed (2–4). Early gastric cancer is defined as gastric cancer that invades no deeper than the submucosa, regardless of lymph node metastasis status (5). The prognosis of early gastric cancer is significantly better than that of advanced gastric cancer, and the 5-year overall survival rate after radical resection is more than 90% (6). However, the prognosis of early gastric cancer with lymph node metastasis is worse than that without lymph node metastasis, and has a higher risk of postoperative recurrence (7). Endoscopic resection is the main treatment besides surgery and has been widely accepted, especially in Asia, where is a high incidence of early gastric cancer, because of the minimally invasive, preservation of gastric function, rapid postoperative recovery and prognosis after curative resection that is not inferior to radical surgical resection (8). Endoscopic resection includes endoscopic mucosal resection (EMR) and endoscopic submucosal dissection (ESD). According to the guidelines of the Japanese Gastric Cancer Association, endoscopic resection is indicated for early gastric cancer with an extremely low possibility of lymph node metastasis (9). Since endoscopic resection does not dissect the perigastric lymph nodes, additional surgery is needed for patients with non-curative endoscopic resection or high risk of lymph node metastasis (9, 10). Therefore, accurate preoperative assessment of lymph node metastases is crucial to the choice of surgical approach.

Lymph node metastasis is an important factor in the prognosis of early gastric cancer and the selection of lymph node dissection. Previous studies have shown that the lymph node metastasis rate of early gastric cancer is about 2%–20% (9, 11). At present, some studies have constructed and validated different predictive models, which mainly include risk factors such as depth of invasion, vascular invasion, neural invasion, degree of differentiation and mixed tissue types (12–15). However, most models were based on the results of postoperative pathology, which is not known preoperatively. Some studies used biomarkers to predict the risk of lymph node metastasis in early gastric cancer. Ma et al. (16) constructed a risk stratification model composed of four mi-RNAs (miR153-3p, miR-708, miR-940 and miR-375). Chen et al. (17) predicted lymph node metastasis based on collagen signaling in the tumor microenvironment. Wang et al. (18) predicted lymph node metastasis by tumor-associated neutrophils (TANS). however, their clinical use was limited by complex detection techniques and lack of confirmation from big data. Inflammation plays an important role in the occurrence, invasion and migration, distant metastasis and chemotherapy resistance of gastric cancer (19–21). However, it remains unclear whether the levels of inflammatory markers in peripheral blood are associated with lymph node metastasis in early gastric cancer. On the other hand, gastroscopy, ultrasound endoscopy and computed tomography (CT) are routine preoperative tests, which allow the surgeon to obtain information about the lesion and the perigastric lymph nodes before surgery. Therefore, the purpose of this study was to analyze the relationship between preoperative clinicopathological data and lymph node metastasis, and to construct a nomogram for preoperatively predicting the risk of lymph node metastasis in early gastric cancer, which guides the formulation of a clinical treatment plan.

## Materials and methods

### Patients

We retrospectively analyzed 292 patients with early gastric cancer who underwent surgery at the Second Affiliated Hospital of Nanchang University from July 2017 to December 2021. The inclusion criteria were as follows: (1). postoperative pathologically confirmed early gastric cancer, (2). radical gastrectomy and standard D2 lymph node dissection were performed. The exclusion criteria were as follows: (1). distant metastases; (2). patients with neoadjuvant therapy; (3). two or more sites of primary gastric cancer; (4). previous history of cancer or remnant gastric cancer; (5). patients with preoperative infection or insufficient evidence of infection but temperature >38 degrees Celsius; (6). patients with hematologic disorders or liver, kidney and cardiac dysfunction; (7). incomplete preoperative clinical information.

### Clinicopathologic characteristics

The clinicopathological data of the patients were obtained from the hospital's electronic health record system. The clinical data mainly included sex, age, history of hypertension, history of diabetes and body mass index (BMI). Meanwhile, we collected the test indicators of patients within one week before surgery, such as tumor markers, hemoglobin, albumin value, prealbumin values and inflammatory markers. The levels of inflammatory markers were divided into high and low groups, according to the best cutoff value of the ROC curve. In addition, AFP, CEA, CA199 and CA125 in this research center were considered abnormal when they were above 8.1 ng/ml, 5.0 ng/ml, 37.0 U/ml and 35.0 U/ml, respectively. According to the results of gastroscopy, the location, maximum diameter, macroscopic features and presence of ulcer of tumor were determined. The tumor location was divided into upper 1/3, middle 1/3 and lower 1/3 of gastric. The macroscopic features were divided into elevated type, flat type and depressed type. According to the presence or absence of ulcers, it was divided into ulcerative and non-ulcerative types. According to the pathological results, the histological types were divided into differentiated type and undifferentiated type; differentiated type included well or moderately differentiated adenocarcinoma, papillary adenocarcinoma and tubular adenocarcinoma; undifferentiated type included poorly differentiated adenocarcinoma, undifferentiated adenocarcinoma, signet ring cell carcinoma and mucinous adenocarcinoma. The preoperative CT results (such as the thickness of the lesion, the presence of perigastric lymph nodes and the maximum short-axis diameter of lymph nodes) were collected. If the maximum short-axis diameter of perigastric lymph nodes was greater than 5 mm, they were considered as enlarged lymph nodes. The CT results were confirmed by two radiologists above the deputy director.

### Statistical analysis

For continuous variables, the normality test was first performed with a single-sample *K-S* test, If the variables conformed to the normal distribution, they were described by mean and standard deviation, and analyzed by *t* test. Otherwise, they were described by median and quartile spacing, and analyzed by the Mann-Whitney *U* test. Categorical variables were described with rates, Chi-square test (or Fisher's exact test in specific conditions) was used for data analysis. The random forest algorithm was used to calculate the importance ranking of meaningful variables in univariate analysis, and these variables were included in multivariate logistic analysis. The stepwise backward regression method was selected to analyze the risk factors of lymph node metastasis in early gastric cancer. A nomogram was constructed according to the results of the multivariate logistic regression model. The predictive ability of the nomogram was evaluated by the C index and ROC curve. The calibration curve and Brier score were used as the indicators of internal calibration. Meanwhile, the Hosmer-Lemeshow test was used to evaluate the goodness of fit of the nomogram. 100 times ten-fold cross-validation was used for internal validation. Finally, in order to measure the clinical practicality, the net benefit was measured by a clinical decision curve. A control model was constructed with the variables of absolute indications or expanded indications for endoscopic resection in the Japan Gastric Cancer Association guidelines, and the models were compared by applying the Net Reclassification Index (NRI) and the Integrated Discriminant Improvement Index (IDI). All of the data were analyzed using the R software (version 4.1.1). *P* values (two-sided) < 0.05 were considered statistically significant in all statistical analyses.

### Ethical approval statement

All procedures performed in studies involving human participants have followed the ethical standards of our institutional research committee and were performed in accordance with the Declaration of Helsinki. As it is a retrospective study, this study was approved by the Ethics Committee of the Second Affiliated Hospital of Nanchang University and was granted an exemption from notification consent.

## Results

### Clinicopathologic features of patients

The clinicopathological characteristics of patients with early gastric cancer are shown in Table 1. Lymph node metastasis was found in 33 (11.3%) of 292 patients with early gastric cancer. The average number of dissected lymph nodes was 22 ± 8. The average age of the patients was 60 years old (28–86 years old), the average maximum diameter of the tumor was 2.24 ± 1.17 cm, the average hemoglobin was 131.93 ± 20.08 g/L, the average albumin was 42.45 ± 3.77 g/L, and the average prealbumin was 254.13 ± 65.56 mg/L. 58.2% of the tumors were located in the lower 1/3 of the stomach. 164 cases (56.2%) in the mucosal (T1a) and 128 cases (43.8%) had invaded to the submucosal (T1b), and the lymph node metastasis rates were 3.1% and 21.9%, respectively. More than 50% of the patients showed thickening of lesions on CT, 52 patients (17.8%) found enlarged lymph nodes on CT, and the rate of lymph node metastasis was 30.8%. The percentages of AFP, CEA, CA199 and CA125 above the normal range were 4.8%, 7.2%, 3.4% and 1.7%, respectively.

**Table 1:** Univariate analysis of preoperative clinicopathological factors.

Variables	Overall	LNM (−)	LNM (+)	*P* value
*N*	292	259	33	
HB [mean (SD)]	131.93 (20.08)	132.60 (19.73)	126.73 (22.22)	0.114
ALB [mean (SD)]	42.45 (3.77)	42.62 (3.71)	41.10 (4.01)	0.028
pALB [mean (SD)]	254.13 (65.56)	255.62 (64.60)	242.44 (72.69)	0.277
Fib [mean (SD)]	2.85 (0.79)	2.84 (0.75)	2.92 (1.07)	0.577
Hypertension (%)				0.069
No	241 (82.5)	218 (84.2)	23 (69.7)	
Yes	51 (17.5)	41 (15.8)	10 (30.3)	
Diabetes (%)				0.910
No	254 (87.0)	226 (87.3)	28 (84.8)	
Yes	38 (13.0)	33 (12.7)	5 (15.2)	
Sex (%)				0.280
Male	180 (61.6)	163 (62.9)	17 (51.5)	
Female	112 (38.4)	96 (37.1)	16 (48.5)	
Location (%)				0.486
Upper third	23 (7.9)	22 (8.5)	1 (3.0)	
Middle third	99 (33.9)	86 (33.2)	13 (39.4)	
Lower third	170 (58.2)	151 (58.3)	19 (57.6)	
Age (%)				0.805
<40	12 (4.1)	10 (3.9)	2 (6.1)	
40–60	129 (44.2)	114 (44.0)	15 (45.5)	
>60	151 (51.7)	135 (52.1)	16 (48.5)	
CT				
Thickness of lesion (%)				0.719
Absence	128 (43.8)	115 (44.4)	13 (39.4)	
Presence	164 (56.2)	144 (55.6)	20 (60.6)	
Enlarged LN, ≥5 mm (%)				<0.001
Absence	240 (82.2)	223 (86.1)	17 (51.5)	
Presence	52 (17.8)	36 (13.9)	16 (48.5)	
Ulcer (%)				0.010
Absence	163 (55.8)	152 (58.7)	11 (33.3)	
Presence	129 (44.2)	107 (41.3)	22 (66.7)	
Morphology (%)				0.028
Elevated type	51 (17.5)	44 (17.0)	7 (21.2)	
Flat type	95 (32.5)	91 (35.1)	4 (12.1)	
Depressed type	146 (50.0)	124 (47.9)	22 (66.7)	
Size (%)				0.007
<2 cm	116 (39.7)	108 (41.7)	8 (24.2)	
2–3 cm	131 (44.9)	117 (45.2)	14 (42.4)	
>3 cm	45 (15.4)	34 (13.1)	11 (33.3)	
T1 (%)				<0.001
1a	164 (56.2)	159 (61.4)	5 (15.2)	
1b	128 (43.8)	100 (38.6)	28 (84.8)	
BMI (%)				0.087
<23.9	206 (70.5)	178 (68.7)	28 (84.8)	
≥24.0	86 (29.5)	81 (31.3)	5 (15.2)	
AFP (%)				1.000
<8.1 ng/ml	278 (95.2)	247 (95.4)	31 (93.9)	
≥8.1 ng/ml	14 (4.8)	12 (4.6)	2 (6.1)	
CEA (%)				0.128
<5.0 ng/ml	271 (92.8)	243 (93.8)	28 (84.8)	
≥5.0 ng/ml	21 (7.2)	16 (6.2)	5 (15.2)	
CA199 (%)				0.016
<37.0 U/ml	282 (96.6)	253 (97.7)	29 (87.9)	
≥37.0 U/ml	10 (3.4)	6 (2.3)	4 (12.1)	
CA125 (%)				0.926
<35.0 U/ml	287 (98.3)	254 (98.1)	33 (100.0)	
≥35.0 U/ml	5 (1.7)	5 (1.9)	0 (0.0)	
FAR (%)				0.055
Low (<0.055)	81 (27.7)	77 (29.7)	4 (12.1)	
High (≥0.055)	211 (72.3)	182 (70.3)	29 (87.9)	
FpAR (%)				0.118
Low (<0.012)	148 (50.7)	136 (52.5)	12 (36.4)	
High (≥0.012)	144 (49.3)	123 (47.5)	21 (63.6)	
NLR (%)				0.040
Low (<2.239)	142 (48.6)	132 (51.0)	10 (30.3)	
High (≥2.239)	150 (51.4)	127 (49.0)	23 (69.7)	
PLR (%)				0.255
Low (<110.254)	82 (28.1)	76 (29.3)	6 (18.2)	
High (≥110.254)	210 (71.9)	183 (70.7)	27 (81.8)	
LMR (%)				0.675
Low (<2.599)	156 (53.4)	140 (54.1)	16 (48.5)	
High (≥2.599)	136 (46.6)	119 (45.9)	17 (51.5)	
SII (%)				0.072
Low (<381.237)	108 (37.0)	101 (39.0)	7 (21.2)	
High (≥381.237)	184 (63.0)	158 (61.0)	26 (78.8)	
SIRI (%)				0.016
Low (<0.458)	72 (24.7)	70 (27.0)	2 (6.1)	
High (≥0.458)	220 (75.3)	189 (73.0)	31 (93.9)	
PNI (%)				1.000
Low (<43.620)	174 (59.6)	154 (59.5)	20 (60.6)	
High (≥43.620)	118 (40.4)	105 (40.5)	13 (39.4)	
Histological type (%)				0.013
Differentiated	161 (55.1)	150 (57.9)	11 (33.3)	
Undifferentiated	131 (44.9)	109 (42.1)	22 (66.7)	

### Predictors for LNM in ECG patients

**Figure 1:**
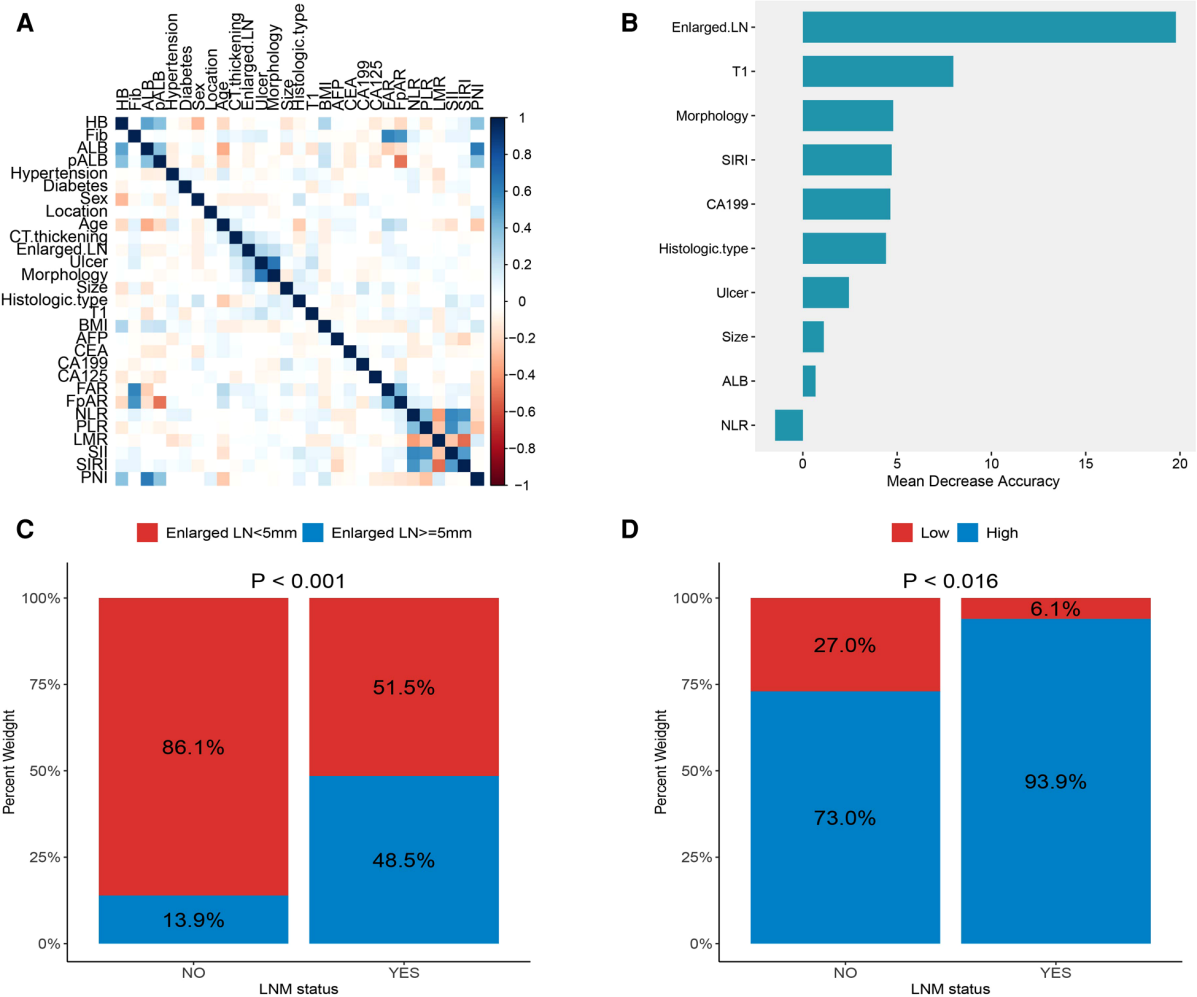
(**A**) correlations between variables; (**B**) the importance ranking of significant variables in univariate analysis; (**C**) the proportion of enlarged lymph nodes found on CT in different lymph node status; (**D**) proportion of systemic inflammatory response index (SIRI) in different lymph node status.

Figure 1A shows the correlations between 29 variables. In univariate analysis, 10 variables were associated with lymph node metastasis in early gastric cancer, which included preoperative albumin level, enlarged lymph nodes on CT, ulcers, macroscopic features, tumor size, depth of invasion, carbohydrate antigen 199 (CA199), neutrophil-to-lymphocyte ratio (NLR), systemic inflammatory response index (SIRI) and histological type (Table 1). The importance of 10 variables was ranked by random forest algorithm, and the results showed that the presence of enlarged lymph nodes on CT and the depth of invasion were important variables for lymph node metastasis (Figure 1B). Multivariate analysis showed that enlarged lymph nodes on CT (OR: 6.765 *P* < 0.001), depth of invasion (OR: 8.622 *P* < 0.001), CA199 (OR: 6.138 *P* = 0.02), systemic inflammatory response index (OR: 4.971 *P* = 0.046) and histological type (OR:3.908 *P* = 0.003) were independent risk factors for lymph node metastasis in early gastric cancer (Table 2).

**Table 2:** Multivariate analysis of preoperative clinicopathological factors associated with LNM.

Factors		OR	95% CI	*P* value
Enlarged LN on CT	Absence	Reference		
	Presence	6.765	2.755–17.361	<0.001
T1	1a	Reference		
	1b	8.622	3.209–28.427	<0.001
CA199	<37.0 U/ml	Reference		
	≥37.0 U/ml	6.138	1.265–28.972	0.020
SIRI	Low (<0.458)	Reference		
	High (≥0.458)	4.971	1.256–33.973	0.046
Histological type	Differentiated	Reference		
	Undifferentiated	3.908	1.619–10.259	0.003

### Development and validation of the nomogram

**Figure 2:**
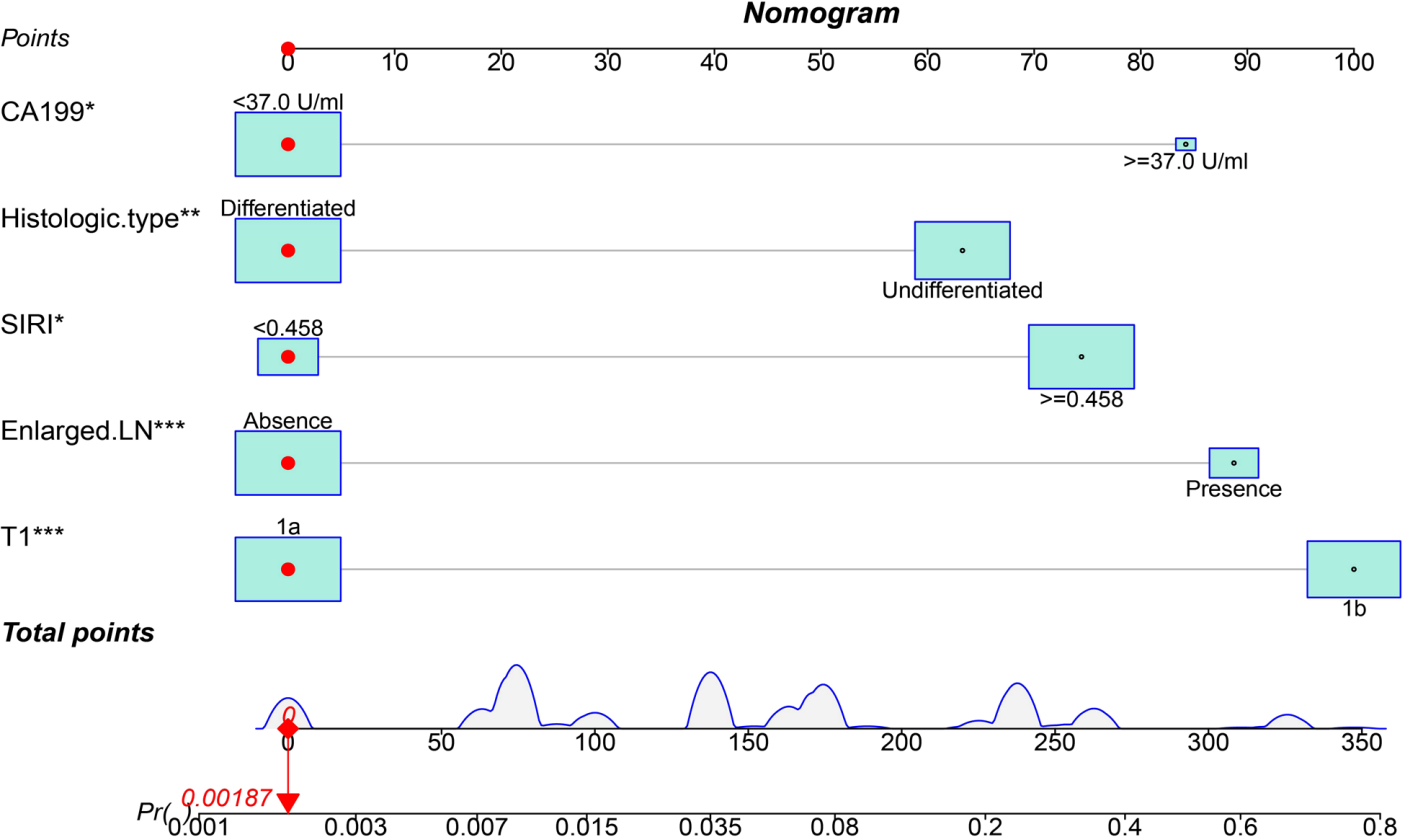
A nomogram for predicting the risk of lymph node metastasis in early gastric cancer. The scores for each variable were summed, and the total score corresponds to the probability of lymph node metastasis.

**Figure 3:**
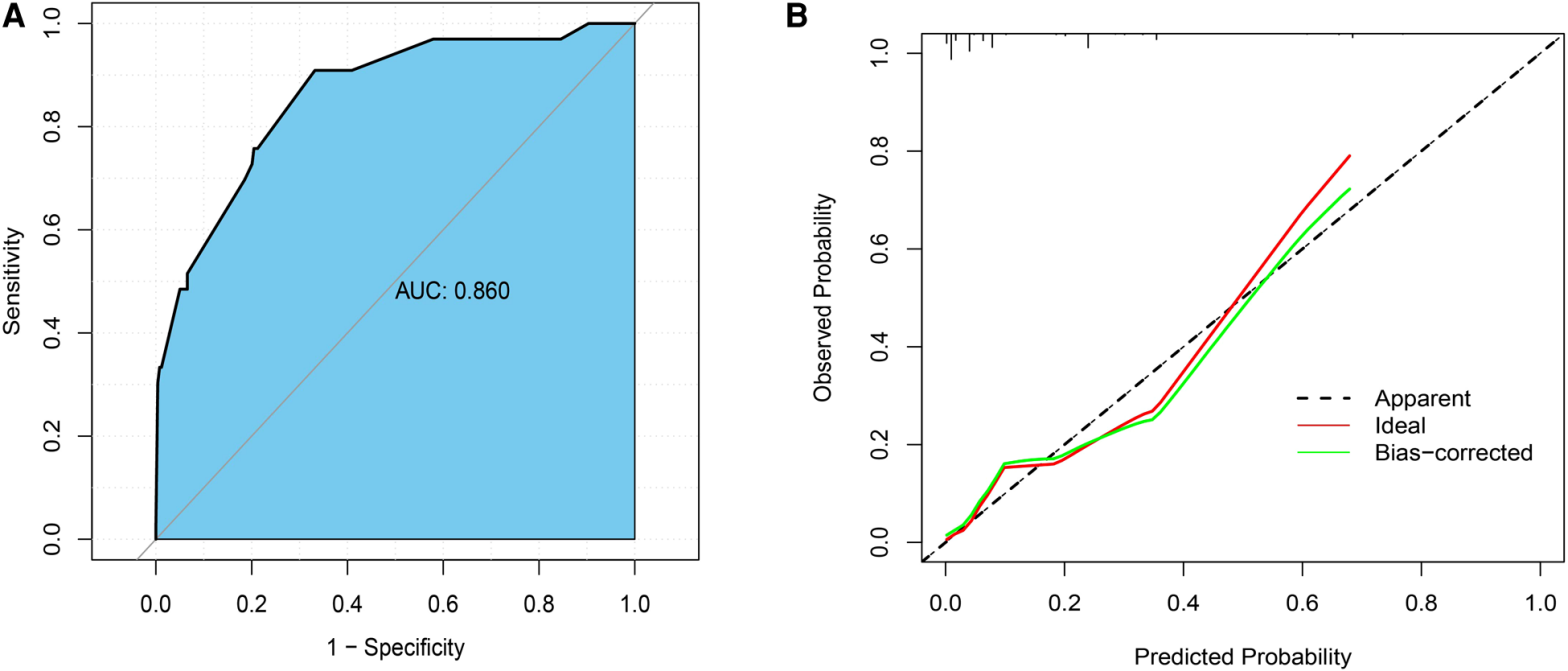
(**A**) the receiver operating characteristic curve (ROC) of the nomogram; (**B**) the calibration curve.

Based on the results of multivariate analysis, we established a nomogram for preoperatively predicting the risk of lymph node metastasis in early gastric cancer. When applying the nomogram, we can calculate an individualized total score for each patient and estimate the risk of lymph node metastasis (Figure 2). The *P* value of the Hosmer-Lemeshow test was 0.60, indicating a good fit of the nomogram. The area under the ROC curve was 0.86, the C-index was 0.86, and the C-index after 1,000 bootstrap corrections was 0.84, which indicated that the nomogram had good discriminative ability (Figure 3A). The calibration curve showed that the nomogram predicts the risk of lymph node metastasis in good agreement with the actual situation (Figure 3B). The Brier score was 0.07, which indicated that the prediction calibration of model is good. The accuracy, sensitivity, specificity, positive predictive value (PPV) and negative predictive value (NPV) of the nomogram were 69.5%, 66.8%, 90.9%, 98.3% and 25.9%, respectively. The nomogram was internally validated by 100 times 10-fold cross-validation. The average area under the ROC curve was 0.85, and the average Brier score was 0.08. These indicators did not change much after cross-validation, which proved that the performance of the nomogram is good and has good generalization ability.

**Figure 4:**
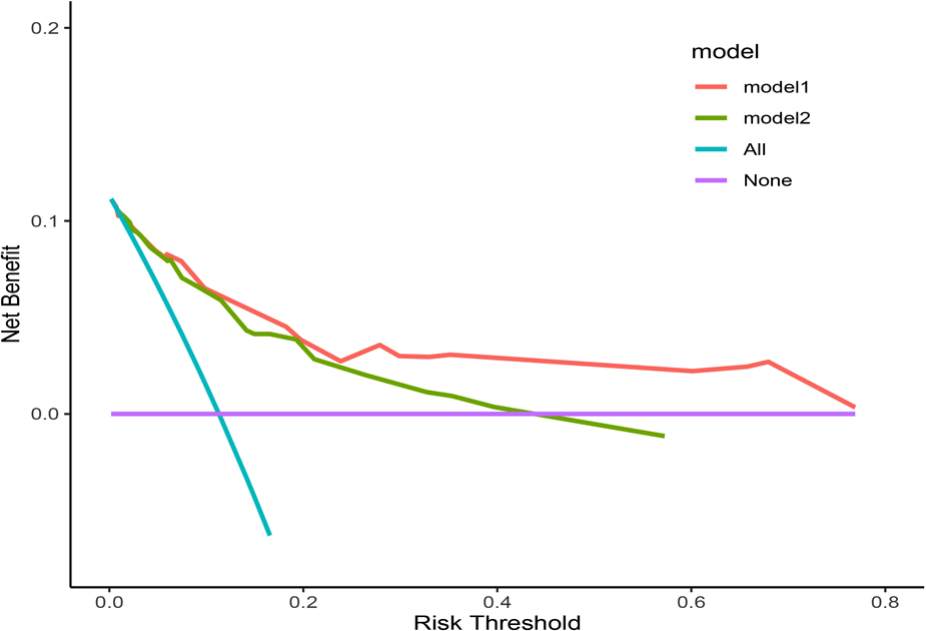
The DCA curve. Model 1 is the nomogram in the study, and Model 2 is the control model, which is composed of variables such as tumor size, histological type, ulcer and invasion depth.

The clinical decision curve (DCA) showed that the nomogram had better clinical utility than the control model (Figure 4) (Table 4). Meanwhile, both the Net Reclassification Index (NRI) and the Integrated Discriminant Improvement Index (IDI) indicated that the nomogram was superior to the control model, and the corresponding *P* value was 0.001 (Table 3).

**Table 3:** Model performance comparison.

	AUC	AUDC	NRI	*P* value (NRI)	IDI	*P* value (IDI)
Model 1	0.860	0.028	0.336	0.001	0.121	0.001
Model 2	0.829	0.015	Reference		Reference	

## Discussion

At present, there are various surgical methods for the treatment of early gastric cancer, including open surgery, laparoscopic surgery, robotic surgery, and endoscopic surgery, but R0 resection is the only effective way to cure early gastric cancer. For EGC patients without lymph node metastasis, endoscopic *en bloc* resection can achieve the curative effect, while patients with non-curative endoscopic resection require additional salvage surgery (10, 22). Therefore, it is particularly important to accurately predict the risk of lymph node metastasis before surgery for the formulation of treatment plans for early gastric cancer.

In this study, a nomogram was constructed to predict the risk of lymph node metastasis in early gastric cancer based on preoperative clinicopathological factors. The lymph node metastasis rate of early gastric cancer in the study was 11.3%, which was consistent with previous studies (9, 11). The study found that tumor invasion into the submucosa, undifferentiated type, carbohydrate antigen CA199 ≥ 37.0 U/ml and enlarged lymph nodes on CT were independent risk factors for lymph node metastasis. Previous studies have confirmed the correlation between these risk factors and lymph node metastasis (23–25). In addition, the study found that the systemic inflammatory response index (SIRI) is also an independent risk factor for lymph node metastasis in early gastric cancer. As far as we know, this is the first study to investigate the relationship between systemic inflammatory response index and lymph node metastasis in early gastric cancer.

Gastric cancer is a highly heterogeneous tumor. Some studies have confirmed that tumor-related inflammation plays an important role in the occurrence, development, treatment response and prognosis of gastric cancer (26, 27). Tumor-related inflammation leads to neutrophilia, thrombocytosis, lymphocytopenia and elevated fibrinogen levels, while the systemic inflammatory state of individuals can be reflected by changes in the levels of leukocytes and fibrinogen in peripheral blood (28, 29). Therefore, the relationship between inflammatory markers in peripheral blood and lymph node metastasis of early gastric cancer was analyzed completely in this study. Univariate analysis showed that neutrophil-to-lymphocyte ratio (NLR) and systemic inflammatory response index (SIRI) were associated with lymph node metastasis, while platelet-to-lymphocyte ratio (PLR), lymphocyte-to-monocyte ratio (LMR), systemic immune inflammatory index (SII), prognostic nutritional index (PNI), fibrinogen to albumin or prealbumin ratio (FAR/FpAR) were not associated with lymph node metastasis, and multivariate analysis showed that only SIRI was a risk factor for lymph node metastasis. Lou et al. (30) confirmed that NLR was associated with lymph node metastasis in early gastric cancer, but only two inflammatory markers (NLR and PLR) were included in the study. Previous studies have confirmed that the higher the systemic inflammatory response index, the later the TNM staging of gastric cancer patients, the poorer prognosis and chemotherapy efficacy, the higher the risk of recurrence, and the predictive performance of the SIRI is better than other inflammatory markers (29, 31–34). In this study, it was found that EGC patients with high SIRI are prone to lymph node metastasis, and the ability of SIRI to predict lymph node metastasis was also superior to other inflammatory markers.

CT scan is a routine examination for preoperative assessment of lymph node status in early gastric cancer, and it is mainly judged that lymph nodes are malignant according to their diameter (35). However, inflammatory reactive lymph nodes are enlarged and smaller lymph nodes may have metastases, which can lead to inaccurate assessment of lymph nodes in some patients (36). Therefore, the appropriate size criteria as an indicator to assess lymph node status remains controversial. Saito et al. (35) reported that the accuracy of CT in assessing lymph node status was about 70%. Wei et al. (23) and Yin et al. (24) showed that the presence of enlarged lymph nodes on CT was a risk factor for lymph node metastasis in early gastric cancer. In this study, the cut-off value of the lymph node diameter was 5 mm, and the diameter > 5 mm was identified as enlarged lymph nodes. The results showed that the accuracy rate and recall rate of CT in evaluating lymph node status were 81.8% and 60.6%, respectively, which also confirmed that enlarged lymph nodes found on CT were independent predictors of lymph node metastasis in early gastric cancer.

The depth of tumor invasion and histological type are also risk factors for predicting lymph node metastasis in early gastric cancer. Previous studies have shown that the incidence of LNM in intramucosal and submucosal of early gastric cancer is 0%–7% and 10%–25% (37), respectively. In this study, the incidence of LNM in intramucosal cancer was 3.1% (5/164), and the incidence of LNM in submucosa cancer was 21.9% (28/128). To a certain extent, the depth of tumor invasion reflects the growth time of the tumor, and the deeper the depth of invasion, the greater the risk of lymph node metastasis. While capillaries are enriched in the mucosal layer, lymphatic vessels are mainly present in the submucosa (38). This phenomenon explains the difference in the incidence of LNM with different depths of invasion. At present, endoscopic ultrasonography has high accuracy in diagnosing the depth of invasion of early gastric cancer. Kim et al. (39) showed that the accuracy of endoscopic ultrasonography for T1a with lesions < 2 cm was 84.6%, and the accuracy of early gastric cancer with lesions > 2 cm was also 83.2%. Therefore, endoscopic ultrasonography is helpful to accurately assess the depth of invasion before surgery. Due to the differences between the results of endoscopic ultrasonography and postoperative pathology, in order to accurately analyze the relationship between the depth of invasion and lymph node metastasis, the depth of invasion diagnosed by postoperative pathology was included in the model in this study. Undifferentiated type and mixed type of early gastric cancer have worse biological behavior and a higher possibility of lymph node metastasis (40, 41). Due to the limited number and shallow sampling of preoperative endoscopic biopsy specimens, there was often a discrepancy between preoperative and postoperative histological results. However, studies have shown that histological differences in early gastric cancer range from 9.4% to 16.3% (42, 43), which is acceptable. Therefore, endoscopy biopsy should be performed with as many sites as possible to improve the accuracy of diagnostic histological types.

Serum tumor markers are widely used in the diagnosis of tumors, assessment of treatment efficacy and disease monitoring. Previous studies have shown that the elevated levels of CA199 and CA724 in EGC patients are closely related to lymph node metastasis, and the elevated levels of CEA and CA125 indicate a poor prognosis of EGC (24, 25, 44). In this study, only CA199 was associated with LNM in EGC.

The nomogram constructed in this study has high specificity and positive predictive value, which provides an effective tool for accurately predicting lymph node metastasis before surgery. The ROC curve and DCA curve show that our model has good discriminative ability and clinical applicability.

This study has some limitations. First of all, our study is a single-center retrospective study with a small sample size, which may have selection bias. The prediction model has only carried out internal cross-validation and not external validation, so multi-center big data is needed for further validation. Secondly, indicators such as Helicobacter pylori infection, carbohydrate antigen 724, peripheral blood circulating tumor cells and interleukin-6 were not included in the study, and the accuracy of the model needs to be further improved.

## Conclusion

We found that systemic inflammatory response index (SIRI) is a novel biomarker for preoperative prediction of lymph node metastasis in early gastric cancer. The nomogram constructed by five preoperative clinicopathological factors can accurately predict the risk of lymph node metastasis of early gastric cancer, and provide guidance for EGC patients to choose the appropriate treatment plan.

## Data Availability

The original contributions presented in the study are included in the article/Supplementary Material, further inquiries can be directed to the corresponding author/s.
